# Size-Dependent Cytoprotective Effects of Selenium Nanoparticles during Oxygen-Glucose Deprivation in Brain Cortical Cells

**DOI:** 10.3390/ijms23137464

**Published:** 2022-07-05

**Authors:** Elena G. Varlamova, Sergey V. Gudkov, Egor Y. Plotnikov, Egor A. Turovsky

**Affiliations:** 1 Institute of Cell Biophysics of the Russian Academy of Sciences, Federal Research Center “Pushchino Scientific Center for Biological Research of the Russian Academy of Sciences”, 142290 Pushchino, Russia; 2 Prokhorov General Physics Institute of the Russian Academy of Sciences, 38 Vavilove st., 119991 Moscow, Russia; s_makariy@rambler.ru; 3 Institute of Biology and Biomedicine, Lobachevsky State University of Nizhny Novgorod, 603105 Nizhny Novgorod, Russia; 4 All-Russia Research Institute of Phytopathology of the Russian Academy of Sciences, Big Vyazyomy, 143050 Moscow, Russia; 5 A.N. Belozersky Institute of Physico-Chemical Biology, Lomonosov Moscow State University, 119992 Moscow, Russia; plotnikov@belozersky.msu.ru; 6 V.I. Kulakov National Medical Research Center of Obstetrics, Gynecology and Perinatology, 117997 Moscow, Russia

**Keywords:** oxygen-glucose deprivation, cell death, cortex, astrocyte, neurons, cell protection, signaling, selenoproteins, selenium nanoparticles, calcium, gene expression

## Abstract

It is known that selenium nanoparticles (SeNPs) obtained on their basis have a pleiotropic effect, inducing the process of apoptosis in tumor cells, on the one hand, and protecting healthy tissue cells from death under stress, on the other hand. It has been established that SeNPs protect brain cells from ischemia/reoxygenation through activation of the Ca^2+^ signaling system of astrocytes and reactive astrogliosis. At the same time, for a number of particles, the limitations of their use, associated with their size, are shown. The use of nanoparticles with a diameter of less than 10 nm leads to their short life-time in the bloodstream and rapid removal by the liver. Nanoparticles larger than 200 nm activate the complement system and are also quickly removed from the blood. The effects of different-sized SeNPs on brain cells have hardly been studied. Using the laser ablation method, we obtained SeNPs of various diameters: 50 nm, 100 nm, and 400 nm. Using fluorescence microscopy, vitality tests, PCR analysis, and immunocytochemistry, it was shown that all three types of the different-sized SeNPs have a cytoprotective effect on brain cortex cells under conditions of oxygen-glucose deprivation (OGD) and reoxygenation (R), suppressing the processes of necrotic death and inhibiting different efficiency processes of apoptosis. All of the studied SeNPs activate the Ca^2+^ signaling system of astrocytes, while simultaneously inducing different types of Ca^2+^ signals. SeNPs sized at 50 nm- induce Ca^2+^ responses of astrocytes in the form of a gradual irreversible increase in the concentration of cytosolic Ca^2+^ ([Ca^2+^]_i_), 100 nm-sized SeNPs induce stable Ca^2+^ oscillations without increasing the base level of [Ca^2+^]_i_, and 400 nm-sized SeNPs cause mixed patterns of Ca^2+^ signals. Such differences in the level of astrocyte Ca^2+^ signaling can explain the different cytoprotective efficacy of SeNPs, which is expressed in the expression of protective proteins and the activation of reactive astrogliosis. In terms of the cytoprotective efficiency under OGD/R conditions, different-sized SeNPs can be arranged in descending order: 100 nm-sized > 400 nm-sized > 50 nm-sized.

## 1. Introduction

The microelement selenium has a pleiotropic effect and has cytoprotective effects on healthy tissues under stress, but, on the other hand, it has an anticancer effect, causing apoptosis. In this regard, selenium is of great interest for use in medicine. Selenium has a relatively high photoconductivity; catalytic activity; oxidative reactions; high piezoelectric, thermoelectric, nonlinear optical properties; and a low melting point. In addition, the electrical conductivity of selenium can be increased by several orders of magnitude when exposed to visible light. All the listed properties of selenium are also preserved in nanoparticles synthesized on its basis. A widely used physical method for obtaining SeNPs is the laser ablation method, which was used to obtain nanoparticles in this study. This method allows for quick creation of high-quality nanoparticles, without any chemical contamination, in one stage [1,2].

Ischemic stroke, resulting from various blood flow disorders, affects millions of people a year worldwide. Stroke therapy methods are significantly limited due to rapid metabolism in the brain and poor transport of most neuroprotectors across the blood–brain barrier [3]. Selenium compounds not only suppress the formation of ROS during ischemia/reoxygenation and hypoxia, but also activate mitochondrial biogenesis and, as a consequence, the level of intracellular ATP and Ca^2+^ homeostasis, as well as promote cell survival in the penumbra zone [4,5]. Selenium nanoparticles can easily penetrate the blood–brain barrier, though they have powerful antioxidant properties against the background of reduced cytotoxicity compared to selenium compounds, which can help protect brain cells from ischemic damage [6,7].

It is this property of selenium pleiotropy that makes it extremely important to study the mechanisms of its action on various tissues in connection with the possibility of obtaining nanoparticles that are optimal in size, shape, stability, and other parameters to achieve the most effective cytoprotective effect on brain tissue. There are features common to nanoparticles of various origins in the implementation of their action depending on the size. It is known that, on the one hand, smaller nanomaterials have greater biological activity, but, on the other hand, they are effective for several hours. Whereas larger nanoparticles have longer lasting effects [8]. The effective size cutoff of the overall structure is 10 nm [9]. The upper limit of the size of nanoparticles can vary, but nanoparticles with a diameter of more than 200 nm activate the complement system and are quickly removed from the bloodstream, accumulating in the liver and spleen [10,11]. With regard to nanoselenium, there are only a few studies on the effect of particle size on cellular physiology. The effect of different-sized SeNPs on brain tissue has not been studied practically at all. There is convincing evidence that SeNPs with a diameter of a micrometer or more are biologically inert [12], while subnanomolar nanoparticles, on the contrary, are extremely toxic [13]. It has been shown that nanoselenium with a diameter of 36 nm is more bioavailable to cells compared to selenite or selenomethionine and enhances the activity of glutathione peroxidases and thioredoxin reductases, providing an antioxidant effect, while having a significantly lower cytotoxicity [12,13,14].

In our previous studies, it was found that SeNPs 50 nm in diameter at doses above 3 μg/mL lead to the induction of apoptosis through the activation of caspase-3 with a higher rate of increase for the process in astrocytes compared to neurons. Exposure to these SeNPs simultaneously activates the expression of genes encoding antiapoptotic proteins and reduces the level of proapoptotic (Bax), which indicates the activation of the neuroprotective signaling cascade along with the caspase signaling pathway of apoptosis [6]. Along with this, the use of nanoparticles with a diameter of 100 nm causes the generation of Ca^2+^ signals in astrocytes through the mobilization of Ca^2+^ ions from the ER upon activation of the phosphoinositide signaling pathway. An increase in [Ca^2+^]_i_ in the astrocyte causes a Ca^2+^-dependent secretion of ATP and a lactate release via Cx43 connexin chemichannels. As a result, paracrine activation of the entire network of astrocytes and suppression of the OGD-induced hyperexcitation of neurons occurs. Prolonged incubation of cerebral cortex cells with SeNPs leads to inhibition of the OGD-induced global increase in [Ca^2+^]_i_ and necrotic death through activation of reactive astrogliosis and expression of the genes encoding cytoprotective proteins [15]. Thus, in view of the revealed differences in the effect of identical concentrations of SeNPs on the intracellular signaling of brain cells, there is a need for a detailed study of the mechanisms of the cytoprotective action of selenium nanoparticles of different sizes during ischemia/reoxygenation.

## 2. Results

### 2.1. The Effect of Selenium Nanoparticles of Various Sizes on the Generation of Ca^2+^ Signals by Astrocytes in the Cerebral Cortex

**Figure 1:**
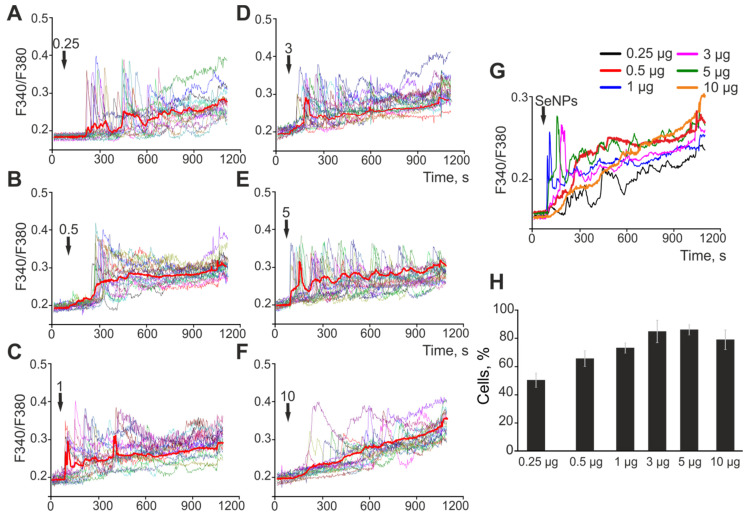
Application of various concentrations of 50 nm-sized SeNPs induces the generation of Ca^2+^ signals in astrocytes of the cerebral cortex. (**A**–**F**)—Ca^2+^ signals of individual astrocytes in one experiment and their average value (red curve) for the application of increasing concentrations of SeNPs. (**G**)—Astrocytic Ca^2+^ signals averaged over several tens of cells for the application of increasing concentrations of 50 nm-sized SeNPs. (**H**)—The percentage of astrocytes that respond with Ca^2+^ signals to the application of SeNPs. The arrow indicates the moment of SeNPs application and their concentration in micrograms per mL (μg/mL).

We have previously shown that the application of SeNPs induces the generation of Ca^2+^ signals exclusively in astrocytes through the mobilization of Ca^2+^ ions from the ER [15]; therefore, below, the Ca^2+^ responses of cerebral cortex astrocytes to the addition of SeNPs of various sizes will be presented. The application of SeNPs with a diameter of 50 nm to the cells of the cerebral cortex at concentrations of 0.25–10 μg/mL (Figure 1A–F) causes a dose-dependent generation of Ca^2+^ signals in astrocytes. In response to the addition of 0.25 μg/mL SeNPs (50 nm), Ca^2+^ signals are generated in 51 ± 7% of astrocytes, and a subsequent increase in the concentration of nanoparticles increases the percentage of responding cells. Ca^2+^ responses in most cases are a transient increase in [Ca^2+^]_i_ without returning the concentration of intracellular Ca^2+^ to the baseline. As can be seen in Figure 1G, Ca^2+^ signals averaged over several tens of astrocytes, upon the addition of any of the studied concentrations of SeNPs, leads to a slow increase in [Ca^2+^]_i_, which may be a sign of impaired Ca^2+^ homeostasis of cells.

**Figure 2:**
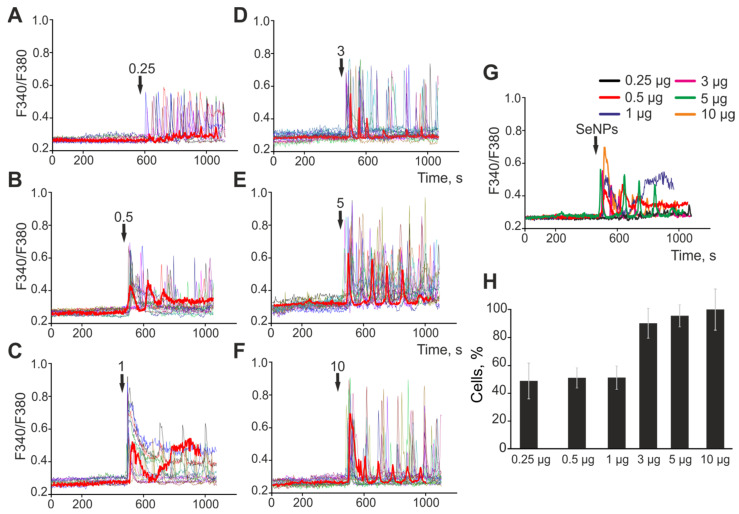
Application of various concentrations of 100 nm-sized SeNPs induces the generation of Ca^2+^ signals in astrocytes of the cerebral cortex. (**A**–**F**)—Ca^2+^ signals of individual astrocytes in one experiment and their average value (red curve) for the application of increasing concentrations of SeNPs. (**G**)—Astrocytic Ca^2+^ signals averaged over several tens of cells for the application of increasing concentrations of 100 nm-sized SeNPs. (**H**)—The percentage of astrocytes that respond with Ca^2+^ signals to the application of SeNPs. The arrow indicates the moment of SeNPs application and their concentration in micrograms per mL (μg/mL).

The application of SeNPs with a diameter of 100 nm also causes the generation of Ca^2+^ signals in astrocytes (Figure 2A–F), and these signals occur in a dose-dependent manner with an increase in the amplitude of Ca^2+^ responses and the percentage (Figure 2H) of responsive cells with an increase in the concentration of nanoparticles. At the same time, the Ca^2+^ responses of cells to the addition of SeNPs (100 nm) are of a cardinally different nature—they are predominantly signals in the form of Ca^2+^ oscillations that occur without an increase in the base level of [Ca^2+^]_i_, which is clearly seen in Figure 2G.

**Figure 3:**
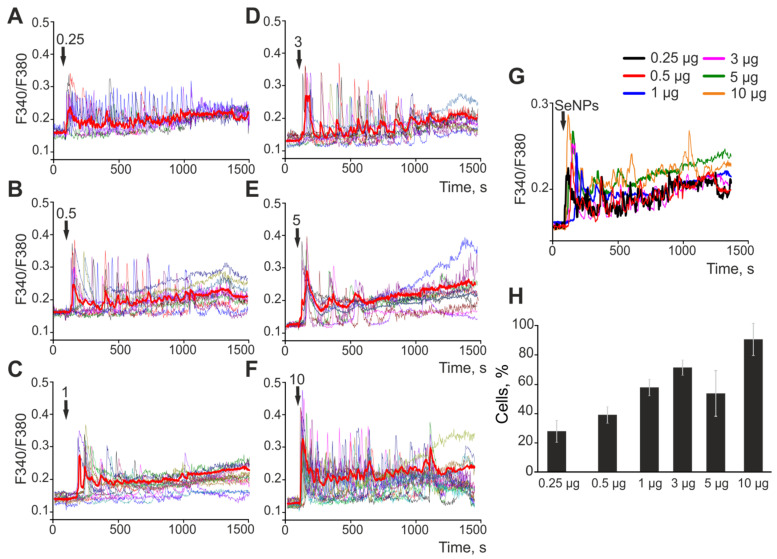
Application of various concentrations of 400 nm-sized SeNPs induces the generation of Ca^2+^ signals in astrocytes of the cerebral cortex. (**A**–**F**)—Ca^2+^ signals of individual astrocytes in one experiment and their average value (red curve) for the application of increasing concentrations of SeNPs. (**G**)—Astrocytic Ca^2+^ signals averaged over several tens of cells for the application of increasing concentrations of 400 nm-sized SeNPs. (**H**)—The percentage of astrocytes that respond with Ca^2+^ signals to the application of SeNPs. The arrow indicates the moment of SeNPs application and their concentration in micrograms per mL (μg/mL).

The largest SeNPs, 400 nm in diameter, added to astrocytes at various concentrations elicit Ca^2+^ responses that also start at a 0.25 μg/mL addition (Figure 3A), and the signal amplitude (Figure 3A–F), simultaneously with the percentage of responding cells (Figure 3H), also increases with increasing concentration. Analysis of the individual responses and averaged Ca^2+^ signals (Figure 3G) showed that the patterns of Ca^2+^ responses of astrocytes to applications of SeNPs (400 nm) are somewhere between 50 nm-sized SeNPs in diameter and 100 nm-sized SeNPs. In particular, most cells react with Ca^2+^ oscillations, similar to the application of 100 nm-sized SeNPs, but these oscillations occur with an increase in the base level of [Ca^2+^]_i_.

Thus, the application of SeNPs of any of the studied diameters leads to a dose-dependent generation of Ca^2+^ signals exclusively in astrocytes of the cerebral cortex; however, the nature of the Ca^2+^ response of cells is significantly different. The addition of SeNPs with a diameter of 50 nm causes predominantly transient Ca^2+^ signals, accompanied by a slow increase in the base level of [Ca^2+^]_i_ during the entire time of fluorescence registration, whereas SeNPs with a diameter of 100 nm cause Ca^2+^ oscillations in astrocytes that occur without an increase in the basic [Ca^2+^]_i_ level. The largest of the investigated nanoparticles (400 nm) lead to the generation of Ca^2+^ oscillations, similarly to the 100 nm SeNPs, but in this case, a moderate increase in the base level of [Ca^2+^]_i_ occurs. Such differences in the behavior of the Ca^2+^ signaling system of astrocytes depending on the size of the SeNPs can lead to different physiological effects, due to the involvement of Ca^2+^ ions in the regulation of most cellular vital processes.

### 2.2. Selenium Nanoparticles of Various Sizes Induce Different Mechanisms of Endocytosis in Astrocytes of the Cerebral Cortex to Generate Ca^2+^ Signals

**Figure 4:**
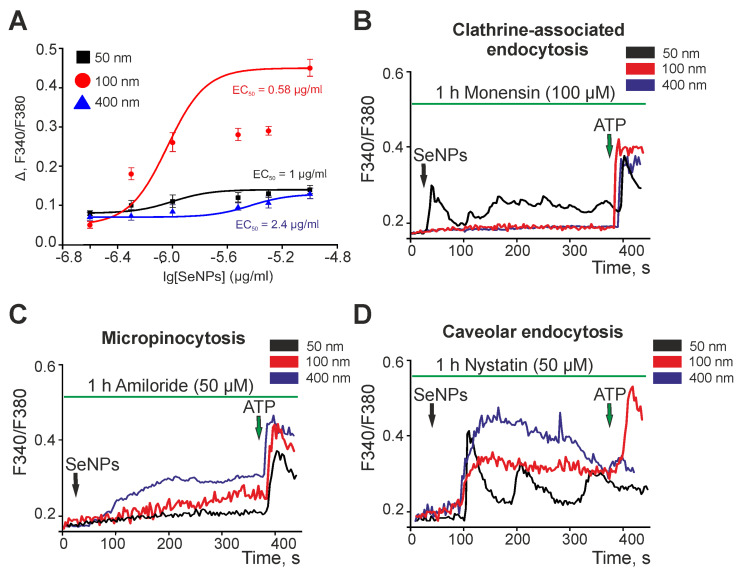
Activation of the Ca^2+^ signaling system of cortical astrocytes by different-sized SeNPs involves different pathways of endocytosis. (**A**)—Dependence of the amplitude of Ca^2+^ responses of astrocytes on the growth concentration of different-sized SeNPs and its approximation by a sigmoid function. (**B**–**D**)—Contribution of clathrin-associated endocytosis (**B**), micropinocytosis (**C**), and caveolar endocytosis (**D**) to the generation of Ca^2+^ signals in astrocytes upon application of 3 µg/mL different-sized SeNPs.

An analysis of the dependence of the amplitude of the Ca^2+^ responses of astrocytes to the application of different-sized SeNPs on their concentration showed that astrocytes of the mouse cerebral cortex are most sensitive to the application of SeNPs with a diameter of 100 nm, since the EC_50_ value for them is 0.58 µg/mL (Figure 4A). Selenium nanoparticles 50 nm and 400 nm in diameter are characterized by transient or vibrational (in the case of 400 nm SeNPs) Ca^2+^ responses, accompanied by a slow increase in the base [Ca^2+^]_i_ concentration. At the same time, EC_50_ for SeNPs 50 nm and 400 nm are 1 μg/mL and 2.4 μg/mL, respectively. Therefore, according to the sensitivity to activation by SeNPs, astrocytes of the cerebral cortex can be arranged in descending order as follows: SeNPs 100 nm > SeNPs 50 nm > SeNPs 400 nm (Figure 4A).

We have previously shown that SeNPs activate the Ca^2+^ signaling system of human glioblastoma cells by activating micropinocytosis and clathrin-associated endocytosis [16]. The application of SeNPs after incubation of astrocytes with monensin, a blocker of clathrin-associated endocytosis, resulted in suppression of the Ca^2+^ signals of cells with the addition of SeNPs with a diameter of 100 nm and 400 nm, while the signals for SeNPs with a diameter of 50 nm were preserved (Figure 4B). The application of different-sized SeNPs against the background of amiloride, a blocker of micropinocytosis, led to complete suppression of the Ca^2+^ signals of astrocytes with the addition of 50 nm-sized SeNPs, and the responses to the addition of large nanoparticles (100 nm and 400 nm) at least changed shape, ceasing to be transient or oscillatory, but were preserved (Figure 4C). The blocker of caveolar endocytosis, nystatin, did not affect the Ca^2+^ responses when SeNPs were added (Figure 4D), which may indicate that this endocytosis pathway does not affect the activation of the Ca^2+^ signaling system of cerebral cortex astrocytes.

Thus, the activation of the Ca^2+^ signaling system of astrocytes in the cerebral cortex occurs more efficiently when using SeNPs with a diameter of 100 nm, as evidenced by the lower value of EC_50_. Such differences in sensitivity to the diameter of SeNPs can be primarily due to the fact that SeNPs of small diameter (50 nm) activate cell Ca^2+^ signals through the only mechanism of endocytosis, clathrin-dependent, while larger SeNPs are simultaneously involved in the generation of Ca^2+^ signals, such as clathrin-dependent endocytosis and micropinocytosis.

### 2.3. Effects of Different-Sized Selenium Nanoparticles on the Survival of Brain Cortex Cells under Conditions of 2-h Oxygen-Glucose Deprivation and 24-h Reoxygenation

**Figure 5:**
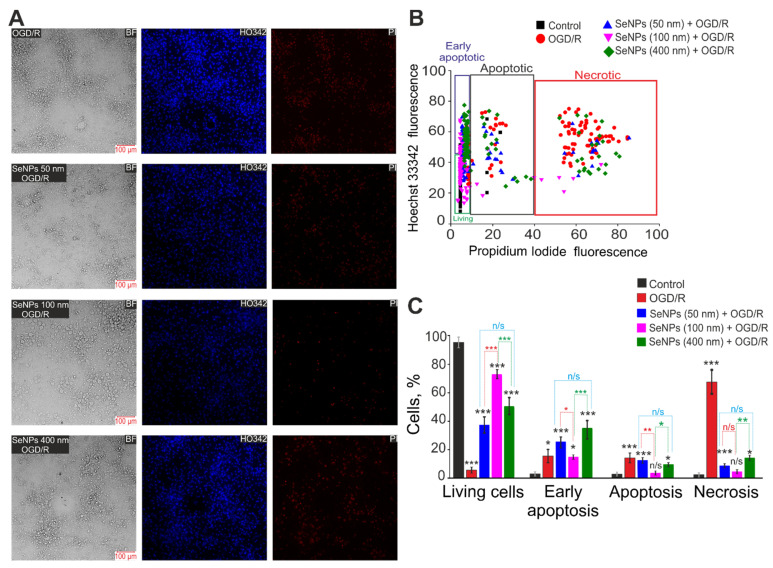
The effectiveness of the cytoprotective action of SeNPs depending on the size on the survival of cerebral cortex cells after 2-h OGD and 24-h reoxygenation (OGD/R). (**A**)—Double staining of cells with Hoechst 33342 (HO342) and Propidium iodide (PI) after 2-h OGD and 24-h reoxygenation. SeNPs’ applications at a concentration of 3 µg/mL were performed 24 h before the OGD/R induction experiments. BF—bright-field microscopy. (**B**)—Cytogram demonstrating the viability of cortical primary cultured neurons and astrocytes. *X*-axis—the intensity of PI fluorescence; *Y*-axis—the intensity of Hoechst 33342 fluorescence. Cells were stained with the probes 24 h after the OGD/R. (**C**)—Effect of 3 μg/mL of different-sized SeNPs on the induction of necrosis and apoptosis 24 h after OGD/R. N cell cultures = 5; *n* coverslips with cells for each sample = 5. For panels D, results are presented as mean ± SEM; n/s—data not significant (*p* > 0.05), * *p* < 0.05, ** *p* < 0.01, and *** *p* < 0.001 compared experimental groups with control (without SeNPs and OGD/R). Comparisons between experimental groups of different sized SeNPs are marked by red (50 nm-sized SeNPs vs. 100 nm-sized), green (100 nm-sized SeNPs vs. 400 nm-sized) or blue (50 nm-sized SeNPs vs. 400 nm-sized) asterisks.

Ischemia-like conditions (oxygen-glucose deprivation, OGD), followed by 24-h reoxygenation, cause a massive death of cortical cells due to the induction of early stages (18%) and late (16%) stages of apoptosis (Figure 5B,C), but mainly necrosis is recorded (63%), detected by the appearance of PI fluorescence (Figure 5A—OGD/R). In the control, without treatment, the deaths of only single cells were detected, no more than 10% of the total number of cells in the microscope’s field of view (Figure 5—Control). Pre-incubation of cells with 3 μg/mL of 50 nm SeNPs reduces OGD/R-induced cell death both by reducing the percentage of apoptosis (22% and 15% in early and late stages, respectively) and by suppressing necrosis (7%) (Figure 5, SeNPs (50 nm) + OGD/R). The addition of 100 nm-sized nanoparticles leads to an even greater decrease in the percentage of cells in the early and late stages of apoptosis (17% and 6%), as well as the suppression of OGD/R-induced necrosis (5%) (Figure 5—SeNPs (100 nm) + OGD/R). However, although it also has a cytoprotective effect on OGD/R-induced cell death, during incubation of cortical cells with SeNPs with a diameter of 400 nm, a redistribution of the percentage of cells is observed: about 38% of cells are registered in the early stages of apoptosis, 12% in the late stages of apoptosis, and the number of necrotic cells increases (17%) (Figure 5—SeNPs (400 nm) + OGD/R), compared with smaller SeNPs. At the same time, it should be mentioned that the addition of 3 μg/mL of different-sized nanoparticles to the culture of the cerebral cortex for 24 h without the application of OGD/R does not cause cell death (Supplementary Figure S1).

**Figure 6:**
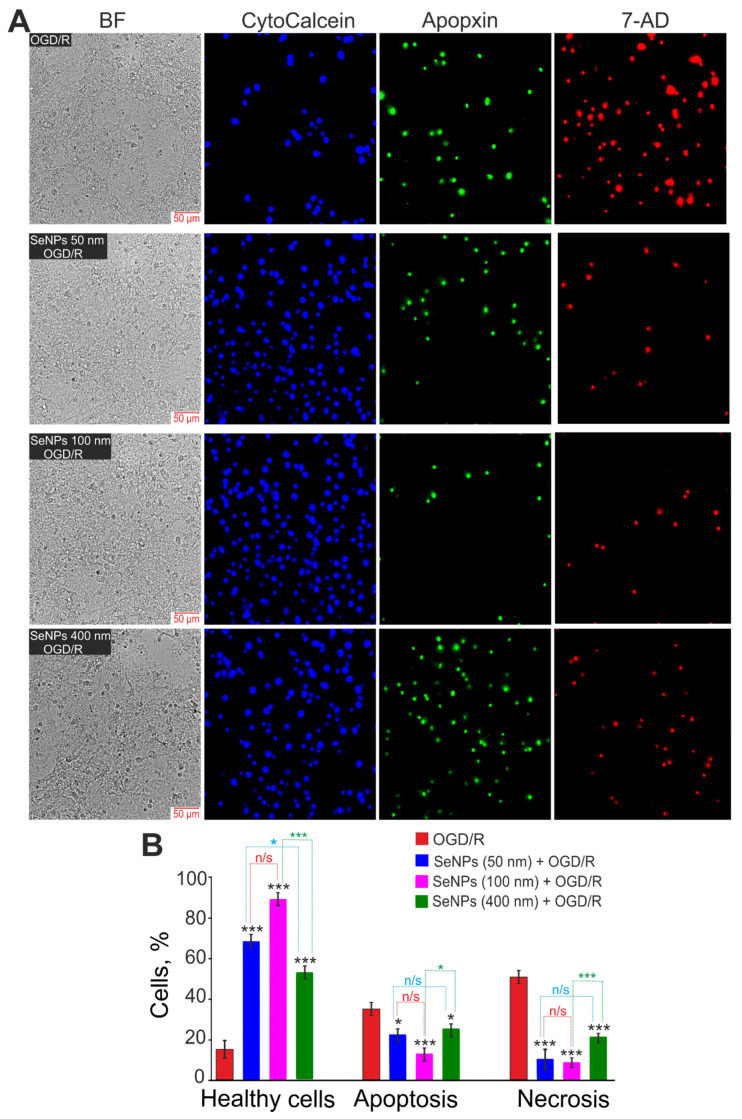
Effect of 24-h incubation of cortical cells with 3 μg/mL of different-sized SeNPs and OGD/R on the induction of necrosis and apoptosis after 24 h OGD/R. (**A**)—Cell staining using the Apoptosis/Necrosis Detection Kit assay. BF—bright-field microscopy, CytoCalcein—healthy cells indicator, Apopxin—apoptotic cells indicator and 7-AD (7-aminoactinomycin D)—necrotic cells indicator. (**B**)—Healthy cells and cells with apoptosis or necrosis after incubation with 3 μg/mL of different-sized SeNPs and OGD/R. Each value is the mean ± SE (*n* ≥ 3). Black asterisks represent comparison of experimental groups versus OGD/R-group. Statistical significance was assessed using paired *t*-test; n/s—data not significant (*p* > 0.05), * *p* < 0.05, and *** *p* < 0.001. Comparisons between experimental groups of different sized SeNPs are marked by red (50 nm-sized SeNPs vs. 100 nm-sized), green (100 nm-sized SeNPs vs. 400 nm-sized) or blue (50 nm-sized SeNPs vs. 400 nm-sized) asterisks.

By staining cells with more specific vital probes, it was possible to confirm the greater efficiency of 100 nm-sized SeNPs in suppressing OGD/R-induced cell death in the cerebral cortex. After 24 h of OGD/R, up to 40% of the cells died along the apoptotic pathway (Apopxin Green staining), and more than 50% of the cells died along the necrotic pathway (staining with 7-aminoactinomycin D) (Figure 6A—OGD/R, Figure 6B). All of the studied different-size SeNPs suppressed OGD/R-induced cell death. However, 100 nm-sized SeNPs are more efficient: 12% of cells die by apoptosis and less than 10% by necrosis (Figure 6).

Thus, all of the studied different-sized SeNPs have a pronounced cytoprotective effect under the conditions of ischemia/reoxygenation, but SeNPs with a diameter of 100 nm have the most pronounced effect, suppressing OGD/R-induced late stages of apoptosis and necrosis to the control level. Larger (400 nm) or smaller (50 nm) nanoparticles were characterized by a high percentage of cells in the early stages of apoptosis, which are considered reversible [17,18].

### 2.4. Effect of 24-h Incubation of Cerebral Cortex Cells with Different-Sized Selenium Nanoparticles on OGD-Induced Increase in [Ca^2+^]_i_

**Figure 7:**
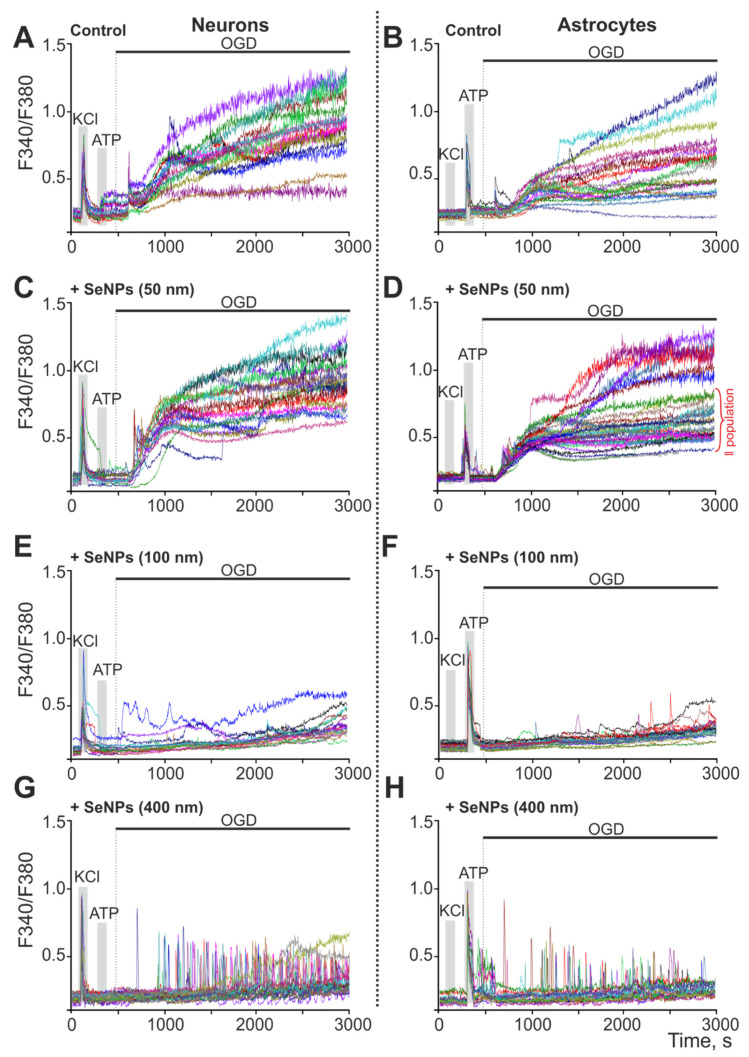
Ca^2+^ signals of neurons and astrocytes on OGD (40 min) in control (**A**,**B**) and after 24-h pre-incubation with SeNPs 50 nm in diameter (**C**,**D**), 100 nm-sized (**E**,**F**), and 400 nm-sized (**G**,**H**). Short-term applications of 35 mM of KCl and 10 µm of ATP were used to detect neurons and astrocytes, respectively. Cellular Ca^2+^ signals in one experiment are shown. The experiments were performed in three repetitions on three separate cell cultures.

It is known that OGD conditions cause Ca^2+^-dependent damage to brain cells, which occurs as a result of a biphasic increase in Ca^2+^ ions in the cytosol of both neurons (Figure 7A) and astrocytes (Figure 7B). It is generally accepted that the second phase of the global increase in [Ca^2+^]_i_ is precisely one of the causes of rapid cell death, which occurs as a result of impaired Ca^2+^ homeostasis. Preliminary (within 24 h) incubation of cerebral cortex cells with 3 μg/mL SeNPs (50 nm) does not lead to inhibition of the first and especially the second phase of the OGD-induced increase in [Ca^2+^]_i_ in neurons (Figure 7C), but, among astrocytes, there is an increase in the number of cells with a lower level of [Ca^2+^]_i_ (Figure 7D—II population). Pre-incubation of cells with 3 μg/mL SeNPs (100 nm) resulted in suppression of the first phase of the OGD-induced [Ca^2+^]_i_ increase in most neurons and an almost complete inhibition of the global [Ca^2+^]_i_ increase (Figure 7F). The same effect completely suppresses Ca^2+^ signals in astrocytes on OGD, and single Ca^2+^ pulses are preserved only in single cells (Figure 7F). The use of large SeNPs (400 nm) also leads to the suppression of the global growth of Ca^2+^ in the cytosol of neurons (Figure 7G) and astrocytes (Figure 7H), but, in both cell types, Ca^2+^ signals on OGD take the form of high-frequency Ca^2+^ oscillations, which in pathological conditions can be regarded as a sign of cell hyperexcitation [19].

Thus, only relatively large SeNPs (100 and 400 nm in diameter) effectively inhibited the global increase in [Ca^2+^]_i_ in neurons and astrocytes during OGD, which led to a more effective decrease in the number of dead cells. Although pre-incubation of cells with 50 nm-sized SeNPs also protected them from OGD-induced death, the cytoprotective mechanism in this case was probably less detectable by Ca^2+^ dynamics.

### 2.5. Comparative Analysis of Expression Patterns of Genes Regulating Cell Death and Ways of Protection against Ischemia Depending on the Size of Selenium Nanoparticles

**Figure 8:**
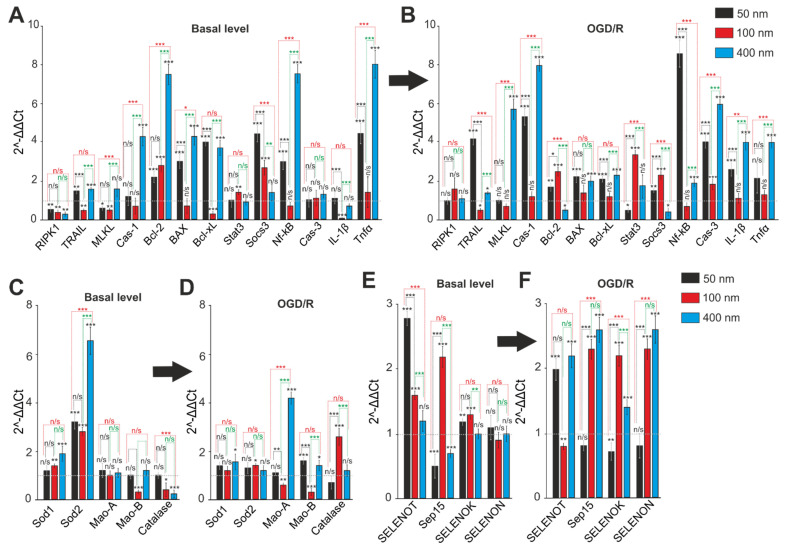
The effect of 24-h incubation of mouse cerebral cortex cells with 3 μg/mL of different-sized SeNPs on the baseline and OGD/R-induced expression levels of genes that regulate cell death, antioxidant status, and selenoproteins. (**A**,**B**)—Basal (**A**) and OGD/R-induced (**B**) levels of expression of genes encoding proteins of necrosis, apoptosis, and inflammation in brain cortex cells after 24-h incubation with 3 μg/mL of different-sized SeNPs. (**C**,**D**)—Basal (**C**) and OGD/R-induced (**D**) levels of expression of genes encoding protein-regulators of antioxidant status in brain cortex cells after 24-h incubation with 3 μg/mL of different-sized SeNPs. (**E**,**F**)—Basal (**E**) and OGD/R-induced (**F**) levels of expression of genes encoding selenoproteins after 24-h incubation with 3 μg/mL different-sized SeNPs. Gene expression in control cells are marked by dashed line for the (**A**,**C**,**E**) panels. Gene expression in OGD/R cells without SeNPs treatment are marked by dashed line for the (**B**,**D**,**F**) panels. Statistical significance was assessed using *t*-test. Comparison of experimental groups regarding control: n/s—data not significant (*p* > 0.05), * *p* < 0.05, ** *p* < 0.01 and *** *p* < 0.001. Comparison of experimental groups relative to each other is indicated in red or green. The number of RNA samples is 5.

After a 24-h incubation of cerebral cortex cells with 3 μg/mL of different-sized SeNPs, the total RNA was isolated, and a PCR analysis of the basic expression of the genes regulating survival pathways (Figure 8A), oxidative status (Figure 8C), and selenoproteins (Figure 8E) was performed. The use of 3 μg/mL SeNPs with a diameter of 50 and 100 nm leads to a decrease in the basic expression of the RIPK1, TRAIL, and MLKL genes responsible for the induction of necrosis, while SeNPs (400 nm) either did not affect the expression level of these genes or increased the expression, especially of the gene Cas-1 encoding Caspase-1 (Figure 8A). The baseline level of the anti-apoptotic genes Bcl-2 and Socs3 significantly increases after incubation of cells with SeNPs 50 and 100 nm, but only in the case of using SeNPs (100 nm) does the expression of the proapoptotic gene Bcl-xL suppression occur. Basic expression of the pro-apoptotic Bcl-xL, BAX, and Nf-kB genes increased many times after cell incubation with 3 µg/mL SeNPs (50 and 400 nm) (Figure 8A). In addition, nanoparticles of this diameter lead to a significant increase in the basal expression of the genes encoding the pro-inflammatory factor Tnfα, while SeNPs 100 nm, on the contrary, reduce its level (Figure 8A).

Of the studied five key genes encoding proteins that regulate the cell redox status, it turned out that the basic level of expression of both forms of Superoxide dismutase (Sod1 and Sod2) increases only in the case of SeNPs with a diameter of 100 nm, while the expression level of Mao-B, on the contrary, decreases. Other SeNPs upregulate Sod2 expression only and do not affect monoamine oxidases (Figure 8C).

As for selenoproteins, incubation of cells with only 3 μg/mL SeNPs (100 nm) resulted in an increase in the baseline expression of three of the four selenoproteins studied (SELENOT, Sep15, and SELENOK), while SeNPs of other sizes had a less pronounced effect (Figure 8E).

It is known that after a 2-h OGD and reoxygenation for 24 h, there is a decrease in the level of expression of genes encoding protective proteins and an increase in the expression of damaging ones [20,21]. Only a pre-incubation of cells with 3 μg/mL SeNPs (100 nm) leads to a decrease in MLKL gene expression and an increase in the level of Bcl-2, Stat3, and Socs3, which is a sign of the anti-apoptotic and anti-necrosis effect of these nanoparticles under OGD/R conditions (Figure 8B). The use of SeNPs with a diameter of 50 nm and 400 nm led mainly to an increase in the expression of pro-apoptotic, and especially pro-inflammatory, genes, while there was no effect on protective genes (Figure 8C). Similarly, after exposure to 100 nm SeNPs, the antioxidant status of cells increases under OGD/R conditions, which is recorded by a significant decrease in the expression of both of monoamine oxidases’ isoforms and an increase in the level of Catalase (Figure 8D). In the case of 50 nm and 400 nm SeNPs, there was a change in the expression patterns of antioxidant status genes in the direction of weakening the antiradical protection of cells. Expression of the genes encoding selenoproteins Sep15, SELENOK, and SELENON increases many times after OGD/R and pre-incubation with SeNPs (100 nm), while the level of SELENOT, on the contrary, decreases by 37%. When using SeNPs with a diameter of 400 nm, the expression of all genes encoding selenoproteins increases, while SeNPs (50 nm) do not significantly affect their expression (Figure 8F).

**Figure 9:**
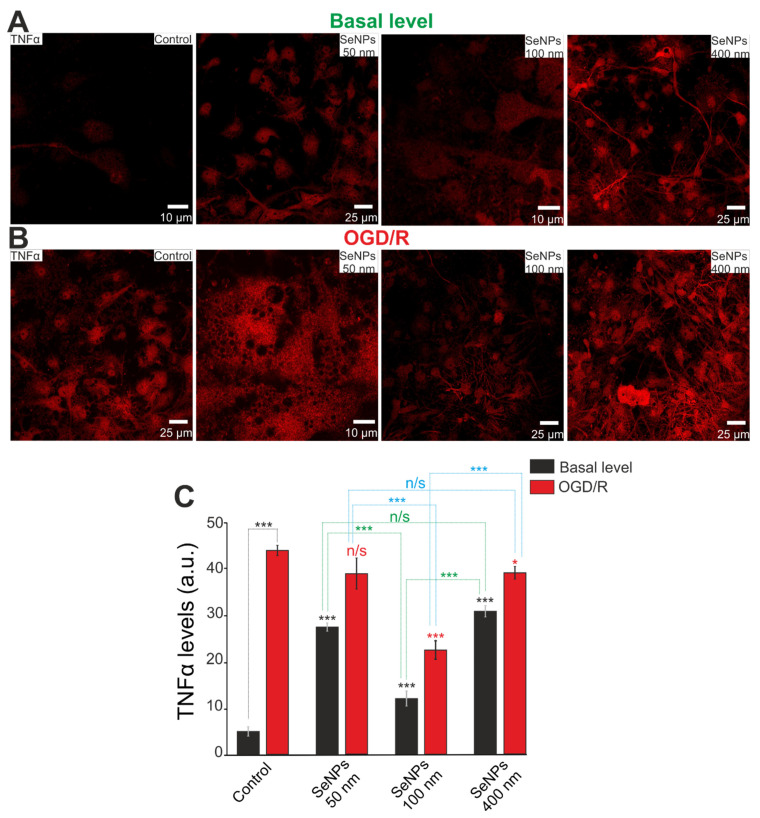
Immunocytochemical staining of cortical cell culture with antibodies against TNFα, a multifunctional pro-inflammatory cytokine, after 24-h incubation with 3 μg/mL of different-sized SeNPs without OGD/R (**A**) and 24 h after OGD/R (**B**). (**C**)—Intensity levels of TNFα were determined by confocal imaging. We analyzed individual cells that had fluorescence of secondary antibodies. The quantative data reflecting the level of TNFα expression are presented as fluorescence intensity values in summary bar charts (mean ± SEM). The values were averaged by 100–150 cells for each column. The results obtained after immunostaining agree with the data of fluorescence presented in panels (**A**,**B**). Each value is the mean ± SE (*n* ≥ 3, *p* < 0.05). Statistical significance was assessed using paired *t*-test. Black asterisks represent comparison of baseline expression levels after incubation with different sized nanoparticles versus control. Red asterisks represent comparison of expression level after incubation with different-sized SeNPs versus OGD/R-induced levels; n/s—data not significant (*p* > 0.05), * *p* < 0.05 and *** *p* < 0.001.

The effects of different-sized SeNPs on the expression of key genes are confirmed by immunocytochemical staining of cell cultures. Figure 9A shows images of cortical cells after 24 h of incubation with SeNPs stained with anti-TNFα antibodies. Baseline TNFα protein increased on average after cell incubation with all SeNPs, but a more pronounced increase was found with 50 nm-sized and 400 nm-sized SeNPs (Figure 9C). After OGD/R, there is a multiple increase in the TNFα levels in cortical cells (Figure 9B); however, pre-incubation with 100 nm-sized and 400 nm-sized SeNPs reduces the OGD/R-induced increase in TNFα (Figure 9C). Moreover, 100 nm-sized SeNPs proved to be the most effective in suppressing inflammation.

**Figure 10:**
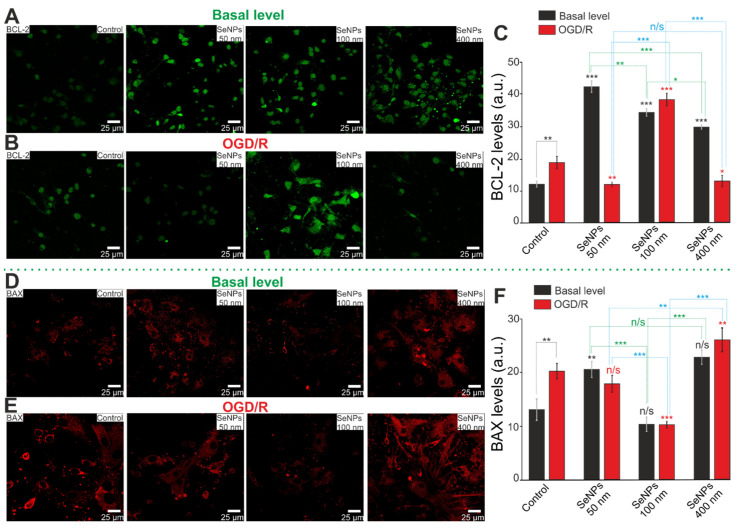
Immunocytochemical staining of cortical cell culture with antibodies against BCL-2 (**A**,**B**) and BAX (**D**,**E**), regulators of apoptosis, after 24-h incubation with 3 μg/mL of different-sized SeNPs without OGD/R (**A**,**D**) and 24 h after OGD/R (**B**,**E**). (**C**,**F**)—Intensity levels of BCL-2 (**C**) and BAX (**F**) were determined by confocal imaging. We analyzed individual cells that had fluorescence of secondary antibodies. The quantative data reflecting the level of BCL-2 or BAX expression are presented as fluorescence intensity values in summary bar charts (mean ± SEM). The values were averaged by 150 cells for each column. The results obtained after immunostaining agree with the data of fluorescence presented in panels (**A**,**B**,**D**,**E**). Each value is the mean ± SE (*n* ≥ 3, *p* < 0.05). Statistical significance was assessed using paired *t*-test. Black asterisks represent comparison of baseline expression levels after incubation with different sized nanoparticles versus control. Red asterisks represent comparison of expression level after incubation with different-sized SeNPs versus OGD/R-induced levels; n/s—data not significant (*p* > 0.05), * *p* < 0.05, ** *p* < 0.01, *** *p* < 0.001.

The balance of the expression level of the key protein regulators of apoptosis BCL-2 and BAX determines cell fate both at rest and under OGD/R conditions. Immunocytochemical staining of cortical cells after 24-h exposure to different-sized SeNPs showed that the baseline level of BCL-2 expression increased after exposure to all SeNPs (Figure 10A,C), which correlates well with the PCR analysis of the Bcl-2 mRNA expression (Figure 8A). At the same time, the basal expression of pro-apoptotic BAX increased after incubation with 50 nm-sized and 400 nm-sized SeNPs, and, after the action of 100 nm-sized SeNPs, the level of BAX did not differ from the control (Figure 10D,F).

Under OGD/R conditions, there is an increase in the content of the BCL-2 protein in control cells, but only 100 nm-sized SeNPs lead to an increase in this protein by three or more times, and SeNPs of other sizes do not affect its level (Figure 10B,C). The level of BAX also increases under OGD/R conditions, and only 100 nm-sized SeNPs reduce OGD/R-induced BAX content, while 50 nm-sized and 400 nm-sized SeNPs, on the contrary, increase BAX in cortical cells (Figure 10E,F).

Thus, pre-incubation of cerebral cortex cells with 3 μg/mL of SeNPs 100 nm in diameter on average leads to a more pronounced decrease in the baseline and OGD/R-induced levels of expression of genes encoding proteins responsible for cell damage and an increase in the expression of protective proteins compared to nanoparticles of other diameters. To a greater extent, the studied nanoparticles affect the expression of genes encoding selenoproteins and the proteins involved in the process of apoptosis and, to a lesser extent, the genes of proteins of the antioxidant status of cells.

### 2.6. The Effect of Different-Sized Selenium Nanoparticles on the Activation of Reactive Astrogliosis under Conditions of Ischemia-Reoxygenation

**Figure 11:**
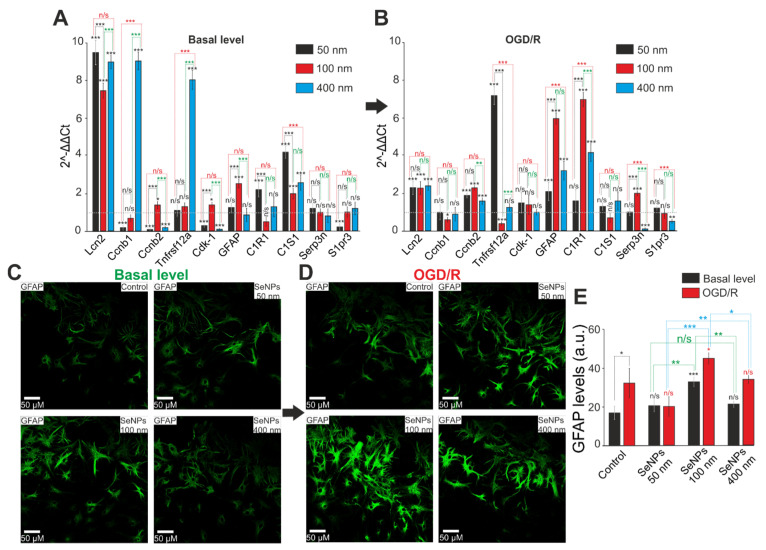
The effect of 24-h incubation of mouse cortical cells with 3 μg/mL of different-sized SeNPs on the activation of basal and OGD/R-induced reactive astrogliosis. (**A**,**B**)—SeNPs (**A**) and OGD/R-induced (**B**) changes in the expression of genes encoding proteins marker of astrocyte reactivity. Dashed line for panels (**A**)—level of gene expression in controls (without treatments). Dashed line for panel (**B**)—level of gene expression in OGD/R cortical cultures. (**C**,**D**)—Immunocytochemical staining of cortical cell culture with astrocytic marker; antibodies against glial fibrillary acidic protein (GFAP) after 24-h incubation with 3 μg/mL of different-sized SeNPs without OGD/R (**C**) and 24 h after OGD/R (**D**). (**E**)—Intensity levels of GFAP were determined by confocal imaging. We analyzed individual cells that had fluorescence of secondary antibodies. The quantative data reflecting the level of GFAP expression are presented as fluorescence intensity values in summary bar charts (mean ± SEM). The values were averaged by 150–200 cells for each column. The results obtained after immunostaining agree with the data of fluorescence presented in panels (**C**,**D**). Each value is the mean ± SE (*n* ≥ 3, *p* < 0.05). Statistical significance was assessed using paired *t*-test. Black asterisks represent comparison of baseline expression levels after incubation with different-sized nanoparticles versus control. Red asterisks represent comparison of expression level after incubation with different-sized SeNPs versus OGD/R-induced levels; n/s—data not significant (*p* > 0.05), * *p* < 0.05, ** *p* < 0.01, *** *p* < 0.001.

Previously, we have shown that SeNPs lead to OGD/R-induced reactive astrogliosis [15]. In this work, we investigated changes in the expression of ten genes encoding key proteins involved in astrocyte reactivation. It turned out that SeNPs with a diameter of 100 nm, after a 24-h incubation, cause an increase in the basic expression of five genes (Lcn2, Ccnb2, Cdk-1, GFAP, C1S1), and SeNPs 400 nm cause an increase in the expression of only four genes (Lcn2, Ccnb1, Tnfrsf12a, C1S1), whereas 50 nm SeNPs lead to a decrease in the baseline expression of four genes and an increase in the expression of only three (Lcn2, C1R1, C1S1) (Figure 11A). Interestingly, only the use of 100 nm SeNPs causes an increase in the expression of the gene encoding the key protein of reactive astrogliosis, GFAP, and this observation is confirmed by immunocytochemical staining of astrocytes (Figure 11C) in which the content of GFAP is higher by 25% only in the group treated with 3 µg/mL SeNPs 100 nm in diameter (Figure 11E).

After OGD/R and incubation with SeNPs (50 nm), the expression of 4 genes out of the 10 studied (Lcn2, Ccnb2, Tnfrsf12a, GFAP) is increased, as well as in the case of SeNPs (400 nm)—Lcn2, Ccnb2, GFAP, C1R1. After 100 nm SeNPs, the expression of five astrogliosis marker genes (Lcn2, Ccnb2, GFAP, C1R1, Serp3n) increases with a significant decrease in the level of Ccnb1, Tnfrsf12a (Figure 11B). Immunocytochemical staining of cell cultures after OGD/R showed that the level of GFAP increased in astrocytes both without treatment with SeNPs and after incubation with SeNPs with a diameter of 100 nm and 400 nm (Figure 11D,E). However, SeNPs (50 nm) do not affect the content of this protein in astrocytes, which is lower even when compared with OGD/R. At the same time, pre-incubation with SeNPs with a diameter of 100 nm turned out to be the most effective for enhancing OGD/R-induced GFAP expression.

Thus, the use of different-sized SeNPs leads to a change in the patterns of basal and OGD/R-induced expression of genes encoding protein markers of reactive astrogliosis. However, the use of SeNPs with a diameter of 100 nm affects a greater number of studied genes and leads to an increase in their expression, especially with regard to the key protein, GFAP. SeNPs with a diameter of 50 nm have the least pronounced effect, leading, among other things, to a decrease in gene expression, but, most importantly, they do not affect the level of the GFAP protein, which may indicate an absence of the effect of these nanoparticles on the activation of astrogliosis of cerebral cortex cells in vitro.

## 3. Discussion

As an important trace element in animals and humans, Se plays an important role in maintaining normal physiological functions of the brain and has a neuroprotective effect. It is known that selenium metabolism in the brain differs from that in other organs because it is predominantly stored in the brain during selenium deficiency [22]. In recent decades, the role of nanoparticles in neurological diseases has been actively studied, since neurons are especially vulnerable to damage caused by oxidative stress for a number of reasons: high oxygen consumption (approximately 25% of the total body intake), the presence of a large amount of polyunsaturated fatty acids, and low levels of antioxidant enzymes [23].

Changes in [Ca^2+^]_i_ concentration regulate almost all intracellular processes and the expression of proteins that will determine a cell’s fate at rest and under stress. Our experiments showed that SeNPs of all studied sizes induce the generation of Ca^2+^ signals; however, the profile of the intracellular increase in Ca^2+^ has a number of fundamental differences. Thus, small (50 nm-sized) SeNPs induce transient Ca^2+^ responses in astrocytes of the cerebral cortex, and the key difference from larger SeNPs is a gradual increase in the base concentration of [Ca^2+^]_i_. SeNPs of a 100 nm size lead to the generation of Ca^2+^ oscillations without a baseline increase in [Ca^2+^]_i_, while the largest SeNPs induce transient Ca^2+^ oscillations with a moderate increase in the baseline [Ca^2+^]_i_ concentration. It is known that an increase in the basic level of [Ca^2+^]_i_ can lead to cell death in the case when such an increase is global and irreversible [21,24] or disturbances in intracellular signaling [25]. In our experiments, the use of 50 nm-sized and 400 nm-sized SeNPs was also accompanied by an increase in the expression of necrosis and apoptosis genes—TRAIL, Cas-1, Bax, Bcl-xL, Nf-kb, and Tnfa—which, however, does not occur without ischemia-caused cell death. SeNPs of this size were less effective as protectors against OGD/R. At the same time, 100 nm-sized SeNPs, which cause Ca^2+^ oscillations without increasing the base level of [Ca^2+^]_i_, on the contrary, increased the expression of protective genes while suppressing pro-apoptotic and pro-necrotic ones. Such a correlation of stable Ca^2+^ oscillations with the activation of protective signaling pathways was shown by us for white adipocytes [26]. Different effects of different-sized SeNPs on the calcium signaling system of cerebral cortex astrocytes and the correlation of Ca^2+^ signals with cell survival under conditions of ischemia/reoxygenation have been shown for the first time. However, there are a sufficient number of works on other nanoparticles that show an increase in toxic properties with a decrease in their size. For example, for chitosan nanoparticles, their cytotoxic effect was shown to depend solely on size, as small-sized nanoparticles increase the death of hematopoietic stem cells [27]. Cell lines A549, HepG2, MCF-7, and CGC-7901 showed that 5 nm-sized AgNPs are more toxic than those sized at 20 nm and 50 nm, causing a significantly more pronounced release of lactate dehydrogenase [28]. At the same time, nanoparticles cause varying degrees of effects on cells of various origins. For silver nanoparticles (AgNPs) smaller than 50 nm, a decrease in the percentage of viable human mesenchymal stem cells after incubation for 1 h at a concentration of 10 µg/mL was shown, while the use of 100 µg/mL of nanoparticles with sizes of 10 and 20 nm did not affect the survival of progenitor human adipose-derived stem cells that differentiate normally even after 24-h incubation [29,30]. In our experiments, the generation of Ca^2+^ signals upon the application of SeNPs of various sizes occurred exclusively in astrocytes, and this mechanism was described in detail in our previous work [15].

Nanoparticle size, shape, and core composition are strong determinants of cellular uptake [31]. It is believed that nanoparticles of various natures have a common tendency: they have the best uptake at a size of about 50 nm and a spherical shape [32]. It is assumed that the process of endocytosis of small nanoparticles is accompanied by a membrane-wrapping process [32,33]. In this case, the enthalpic limit for a spherical nanoparticle occurs at a size of about 30 nm, indicating that nanoparticles smaller than this limit will not be able to drive the membrane-wrapping process effectively. Large nanoparticles drive the membrane-wrapping process by binding to many receptors; however, a nanoparticle above 60 nm in diameter results in a receptor shortage, which decreases uptake because of the increasing entropic penalty [3]. Interestingly, the positively charged nanoparticles showed the most absorption at 58 nm, while the negatively charged nanoparticles showed the most absorption at 40 nm. For AgNPs, different activity of endocytosis of nanoparticles of different sizes into cells was shown when cytochalasin D inhibited 10 nm nanoparticles by 35% and 75 nm-sized nanoparticles by 51%. These data show the different involvements of the mechanism of actin-dependent endocytosis when using particles of different sizes. In both cases, endocytosis occurs as a result of the activation of active mechanisms, when the active endocytosis was reduced to 2.4% and 6.8% for 10 nm and 75 nm nanoparticles, respectively, and the medium temperature was lowered to 4 °C, respectively [34]. BEAS-2B cells show that caveolin/lipid raft-mediated endocytosis, nystatin, has virtually no effect on the endocytosis processes of both 10 nm and 75 nm-sized AgNPs, while amiloride, a blocker of macropinocytosis, was most effective in suppressing the uptake of 75 nm-sized nanoparticles. Further, endocytosis of 10 nm AgNPs was most effectively suppressed by amantadine and wortmannin, blockers of clathrin-mediated endocytosis and general fluid-phase endocytosis, respectively [34]. In our experiments with SeNPs, the caveolar pathway of endocytosis was also not observed, since nystatin did not lead to inhibition of astrocyte Ca^2+^ responses. Our results are in good agreement with the above trends. Activation of the Ca^2+^ signaling system of astrocytes occurs more efficiently when using 100 nm-sized SeNPs, as a significantly lower concentration of nanoparticles is required to induce Ca^2+^ responses. This effect is due to the simultaneous activation of two pathways of endocytosis—clathrin-dependent endocytosis and micropinocytosis. Moreover, when small (50 nm) SeNPs are applied, endocytosis occurs through a clathrin-dependent mechanism. However, despite the activation of two pathways of endocytosis, SeNPs with a diameter of 400 nm are the least effective in activating the Ca^2+^ signals of astrocytes, which can be explained by their physical properties and reduced ability to penetrate into cells. For AgNPs, it was shown that approximately 10% of the total amount of Ag in cells existed in the form of Ag ions, and, before the use of nanoparticles, the proportion of Ag ions was 5.2%; the conversion of AgNPs into Ag ions occurs intracellularly [35]. By analogy, a similar situation can be assumed for SeNPs, when smaller selenium nanoparticles (50 nm), penetrating into cells, release a larger amount of selenium ions, leading to some toxic effects on brain cells. At the same time, large silver nanoparticles form agglomerates, reducing the efficiency of endocytosis and the amount of released intracellular silver [34], which can also occur in the case of our SeNPs (400 nm) and lead to less cytoprotective effects. It is known that, along with active endocytosis, under the action of nanoparticles, passive transport into cells is also realized—through passive penetration [36,37]. This transport of nanoparticles is limited to about 200 nm [38], and this process is probably suppressed upon application of large SeNPs.

It is well known that disturbances in the Ca^2+^ homeostasis of brain cells accompany not only neurodegenerative diseases, but also cause the induction of necrosis and apoptosis during ischemia/reoxygenation [15,39]. At the same time, SeNPs are actively involved in the regulation of the Ca^2+^ homeostasis of brain cells and, first of all, astrocytes [40,41,42]. Vitality tests after 2 h of OGD and 24 h of reoxygenation showed that all studied different-sized SeNPs have a cytoprotective effect, significantly reducing the percentage of necrotic cells and reducing the percentage of cells in the late stages of apoptosis. However, only the preincubation of cells with 100 nm-sized and 400 nm-sized SeNPs correlated with an almost complete suppression of the OGD-induced global [Ca^2+^]_i_ increase, and small SeNPs only increased the percentage of cells with a reduced global [Ca^2+^]_i_ increase. At the same time, PCR analysis showed suppression of the baseline and OGD/R-induced expression of pro-apoptotic genes using 100 nm-sized SeNPs, which is explained by Ca^2+^-dependent activation of protective genes. In addition, during ischemia/reoxygenation, ER stress occurs and, as a result, improper folding of selenium-containing proteins and increased cell death, while SeNPs are able to significantly suppress ER stress, which may be another mechanism of their neuroprotective action [43,44]. The mechanism of ER stress involves a number of ER resident selenoproteins, SELENOT, SELENOK, SELENON, SEP15, whose expression levels are regulated by the addition of SeNPs [43,44,45]. Astrocytes have been shown to be able to increase the expression of antioxidant selenoproteins during brain injury during ischemia [46]. When using 100 nm-sized SeNPs, an increase in both the baseline and OGD/R-induced expression of the above selenoproteins was observed, while, when exposed to 400 nm-sized SeNPs, a similar increase was observed only after OGD/R. The use of 50 nm-sized SeNPs in terms of increasing the expression of selenoproteins is practically ineffective. It has been shown that the main role of SELENOK in EPR is the maintenance of calcium homeostasis, and it is the use of large nanoparticles that leads to the suppression of the OGD/R-induced global increase in [Ca^2+^]_i_. Thus, it was shown that SELENOK can interact with palmitoyltransferase (DHHC6) and regulate Ca^2+^ ion fluxes in the ER through IP3R palmitoylation [47,48]. Suppression of SELENOK expression is known to significantly aggravate HepG2 cell death and apoptosis [49]. On the contrary, overexpression of SELENOK protects brain cells from apoptosis due to its antioxidant properties [50]. SELENOT in the brain is responsible for calcium homeostasis, performs antioxidant functions, and controls protein processing in the ER [51]. It has been shown that a decrease in SeLENOT expression in transgenic cell and animal models contributes to the accumulation of reactive oxygen and nitrogen species, Ca^2+^ depletion, activation of the unfolded protein response (UPR), and impaired hormone secretion [52]. Sep15 and SELENON are highly expressed in the brain, and their functions are the least understood of all known selenoproteins. It is known that Sep15 is involved in the induction of adaptive ER stress and has redox activity [53]. SELENON modulates Ca^2+^ homeostasis in brain cells through interaction with the ryanodine receptor, which is also activated in brain cells during ischemia [54]. An increase in the expression of these proteins may also be involved in the mechanism of the cytoprotective action of large SeNPs. In addition, one of the advantages of 100 nm-sized SeNPs is their physical limitation of the possible toxic effect on cells. It has been shown that silver nanoparticles cause predominantly cytotoxic effects, whereas SeNPs, depending on their size, are able to function as intracellular scavengers of free radicals [55]. Toxicological studies have shown that small selenium nanoparticles are characterized by a larger surface area and those particle numbers per unit mass can induce harmful respiratory damage and inflammation. Larger SeNPs (about 100 nm) can be taken up by macrophages, and their aggregation can reduce the toxicity of nanoparticles [56]. It is known that in response to various pathological stimuli, astrocytes can undergo changes in transcriptional regulation, as well as biochemical, morphological, metabolic, and physiological remodeling, characterized by the general name “reactive astrogliosis” [57,58]. Another mechanism of the cytoprotective action of SeNPs is the induction of reactive astrogliosis under the action of ischemia [15,42]. It turned out that 100 nm-sized SeNPs are the most effective in inducing basal and OGD/R-induced reactive astrogliosis by increasing expression of the key genes that regulate this process and increasing the level of GFAP compared to nanoparticles of other sizes.

## 4. Materials and Methods

Experimental protocols were approved by the Bioethics Committee of the Institute of Cell Biophysics. Experiments were carried out according to Act708n (23 August 2010) of the Russian Federation National Ministry of Public Health, which states the rules of laboratory practice for the care and use of laboratory animals, and the Council Directive 2010/63 EU of the European Parliament on the protection of animals used for scientific purposes.

### 4.1. Preparation and Characterization of Selenium Nanoparticles

**Figure 12:**
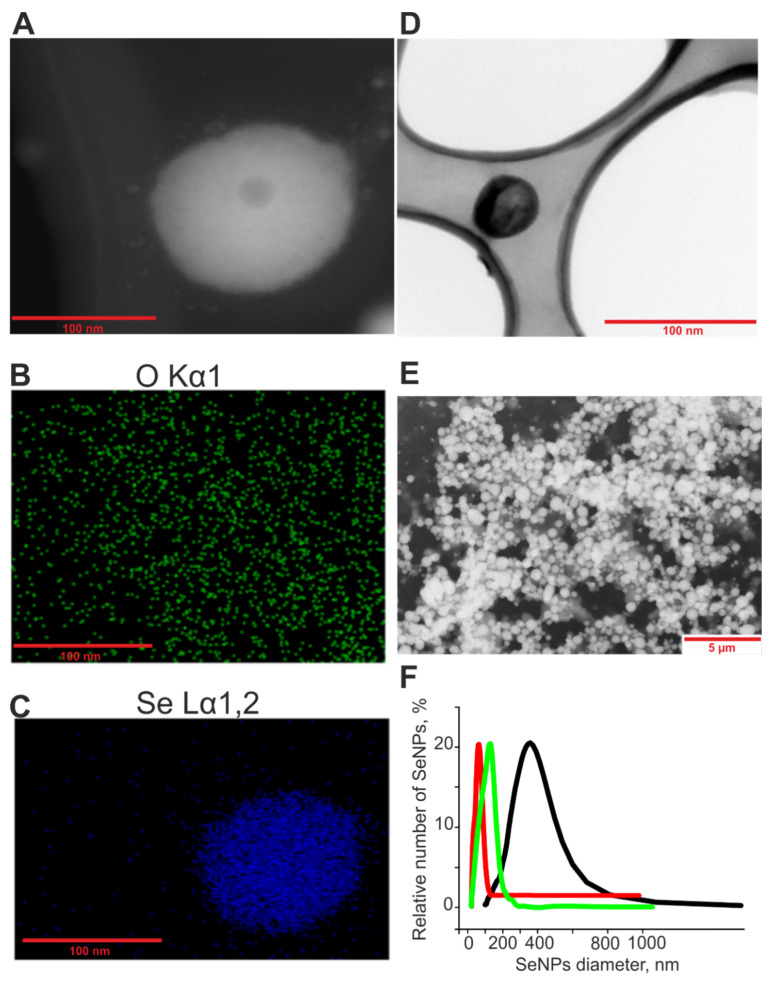
Shape, chemical composition, and size distribution of SeNPs. (**A**)—TEM image of an individual nanoparticle with a diameter of about 100 nm (dark field image). (**B**)—The distribution of oxygen atoms in panel (**A**). (**C**)—The distribution of selenium atoms in panels A. (**D**)—TEM image of an individual nanoparticle with a diameter of 50 nm (bright field image). (**E**)—TEM image of a preparation of nanoparticles with an average diameter of about 400 nm (dark field image). (**F**)—Size distribution of selenium nanoparticles – 50 nm-sized (red line), 100 nm-sized (green line) and 400 nm-sized (black line). Data obtained using an analytical disk centrifuge and confirmed by DLS. PDI_50nm_ = 0.25 ± 0.3; PDI_100nm_ = 0.29 ± 0.5; PDI_400nm_ = 0.34 ± 0.8 (*n* = 3).

The method of obtaining and characterizing SeNPs was described in detail in our previous work [45]. Briefly, SeNPs of various sizes were obtained by laser ablation in deionized water with a resistivity of 18 MΩcm. A solid target was placed at the bottom of the cuvette under a thin layer of water (no thicker than 2–3 mm). A solid target was irradiated with a laser beam through a thin layer of water (λ = 510.6 nm; T = 30 ns; f = 5 kHz; P = 3 kW; Ep = 3 mJ). The mixing of the laser beam on the target was carried out along a given trajectory in the form of parallel straight lines inscribed in a rectangle using an LScanH galvanomechanical scanner (Ateko-TM, Moscow, Russia) [59]. Depending on the characteristics of laser radiation, such as the speed and trajectory of the laser beam, it is possible to obtain colloidal solutions of SeNPs with specified geometric parameters. The size of the nanoparticles was characterized using a DC24000 analytical centrifuge (CPS Instruments, Prairieville, LA, USA). Nanoparticles’ concentrations and hydrodynamic radii were evaluated using Zetasizer Ultra Red Label (Malvern, UK) [60]. It was found that the resulting preparation of SeNPs has a monomodal size distribution (Figure 12). The zeta potential of nanoparticles is about −30 mV (D_50_nm = −31 mV; D_100_nm = −33 mV; D_400_nm = −28 mV). The method of energy dispersive X-ray spectroscopy confirms that the nanoparticles consist of zero-valent selenium. Transmission electron microscopy was performed using a Libra 200MC (Carl Zeiss NTS GmbH, Oberkochen, Germany).

### 4.2. Preparation of Mixed Neuroglial Cell Cultures

Cell cultures from a cerebral cortex were prepared as described in detail previously [6]. Briefly, 0–1-day-old pups were euthanized and decapitated. The extracted tissue was washed with Mg^2+^- and Ca^2+^-free Versene solution and minced with scissors. Then, the tissue fragments were digested with 1% trypsin solution for 10 min at 37 °C and washed two times with cold Neurobasal-A medium. Trypsinized tissue was gently triturated with a pipette, and the debris was then carefully removed with a pipette tip. The obtained cell suspension was seeded on polyethyleneimine-coated glass coverslips and grew for 10 days in vitro in the cell culture medium composed of Neurobasal-A medium, supplement B-27 (2%), and 0.5 mM glutamine.

The drugs were added into the culture medium under sterile conditions in the case of experiments with 24-h pre-incubation with SeNPs. Then, the cell cultures were washed after the pre-incubation with Hank’s balanced salt solution and used in experiments.

### 4.3. Fluorescent Ca^2+^ Measurements

To detect the changes in [Ca^2+^]_i_, cell cultures were loaded with Fura-2 (4 µM; 40 min incubation; 37 °C). The cells were stained with the probe dissolved in Hank’s balanced salt solution (HBSS) composed of (mM): 156 NaCl, 3 KCl, 2 MgSO4, 1.25 KH2PO4, 2 CaCl2, 10 glucose, and 10 HEPES, pH 7.4. To measure [Ca^2+^]_i_, we used the system based on an inverted motorized microscope Leica DMI6000B with a high-speed monochrome CCD-camera HAMAMATSU C9100. For excitation and registration of Fura-2 fluorescence, we used an FU-2 filter set (Leica, Wetzlar, Germany) with excitation filters BP340/30 and BP387/15, beam splitter FT-410, and emission filter BP510/84. Illuminator Leica EL6000 with a high-pressure mercury lamp was used as a source of excitation light. To distinguish between neurons and astrocytes, we used short-term applications of 35 mM KCl and 10 µM ATP before the main experiments. This method was described in detail in our previous work [61]. Briefly, KCl induces depolarization of excitable cells, which contain a wide range of voltage-gated cation channels. KCl-induced depolarization promotes the opening of voltage-gated calcium channels in neurons (predominantly L-type channels). The conductivity and density of the cation channels in astrocytes are insufficient to evoke a high-amplitude Ca^2+^ response to the KCl application. All the Ca^2+^ signals are presented as a 340/380 ratio of Fura-2 fluorescence.

### 4.4. The Technique for Simulation of Ischemia-Like Conditions

Ischemia-like conditions (oxygen-glucose deprivation, OGD) were obtained by omitting glucose (HBSS medium without glucose) and by displacement of dissolved oxygen with argon in the leak-proof system [21]. The level of oxygen in the medium was measured using a Clark electrode. Oxygen tensions reached values of 30–40 mm Hg or less within 20 min of the beginning of displacement. Ischemia-like conditions lasting for 40 min or 2 h were created using supplying the oxygen-glucose deprivation (OGD) medium into the chamber with cultured cortical cells. Constant argon fed into the experimental chamber was used to prevent contact between the OGD-medium with the atmospheric air.

### 4.5. Assessment of Cell Viability and Apoptosis

Propidium iodide (1 µM) was used to evaluate the number of dead cells in the cell cultures before and after OGD. The cells were stained for 5 min with the probes diluted in HBSS and then rinsed with HBSS. Fluorescence of the probes was detected with an inverted fluorescent microscope Zeiss Axio Observer Z1 using Filter Set 20. Cell death induced by OGD was assessed by propidium iodide staining (PI, 1 µM) before and after the exposures in the same microscopic field. Since PI stains both dead astrocytes and neurons, an analysis of calcium signals upon a 35 mM KCl application before OGD was used to identify the type of cells. Neurons were identified by the fast-transient calcium signal upon KCl application, as described previously. Furthermore, we used the Ca^2+^ signals (presence or absence of a global increase in [Ca^2+^]_i_ during OGD) as an additional indicator of cell viability [62].

Hoechst 33342 (2 µM) and propidium iodide (1 µM) were used to evaluate the number of dead cells in the cell cultures before and after 2-h OGD and 24-h reoxygenation. The cells were stained for 5 min with the probes diluted in HBSS and then rinsed with HBSS. Fluorescence of the probes was detected with an inverted fluorescent microscope Zeiss Axio Observer Z1 using Filter Set 01 and Filter Set 20. Discrimination of early and late apoptotic cells was performed according to the previously described method [63]. Five different areas of each cell culture were analyzed. Each experimental group consisted of three cell cultures from different passages.

To simultaneously monitor apoptotic and healthy cells after different-sized SeNPs treatment and OGD/R with fluorescence microscope, an Apoptosis/Necrosis Detection Kit was used. Cells were washed 1–2 times and resuspended with Assay Buffer. To detect apoptotic cells, Apopxin Green Indicator was used. Apoptotic cells were visualized using the FITC channel (Ex/Em = 490/525 nm). For staining necrotic cells, we used 7-aminoactinomycin D (Ex/Em = 550/650 nm). To detect healthy cells, CytoCalcein 450 was used, and cells were visualized using the violet channel (Ex/Em = 405/450 nm).

### 4.6. Immunocytochemical Method

To detect GFAP, TNFα, BCL-2, and BAX in cells, we used an immunocytochemical assay. The cells were fixed with 4% paraformaldehyde +0.25% glutaraldehyde in PBS for 20 min and washed three times with ice-cold PBS for 5 min. Glutaraldehyde was added into the fixative solution to minimize the washing of antibodies from cells during permeabilization. To permeabilize cells, we used 0.1% Triton X-100 solution for 15 min. Fixed cells were incubated in 10% donkey serum for 30 min at room temperature to block non-specific antibody binding sites. The cells were then incubated with primary antibodies against the investigated proteins for 12 h at 4 °C. The fixed cells were subsequently washed with PBS (3 times for 5 min) and probed with secondary antibodies conjugated with a fluorescent label. We used purified monoclonal anti-GFAP antibody (BioLegend, RRID: AB_2632644), monoclonal anti-TNFα antibody (C-4) (Santa Cruz Biotechnology, sc-133192), monoclonal anti-BCL-2 antibody (Thermo Fisher Scientific, MA5-11757, RRID: AB_10978135), and monoclonal anti-BAX antibody (Invitrogen, MA5-14003, RRID: AB_10979735), as well as donkey polyclonal secondary antibody to mouse IgG-H&L (Alexa Fluor-594) (Abcam, RRID: AB_2732073). Dilutions of primary and secondary antibodies were performed according to the manufacturer’s recommendations for immunocytochemical staining. The fluorescence of the antibodies was visualized with an inverted confocal microscope Leica TCS SP5 (Leica, Wetzlar, Germany). Registration of the secondary antibodies’ fluorescence for the control and experimental groups of cell cultures was carried out at the same microscope setting. Fluorescence analysis was performed in ImageJ 2002 software (RRID: SCR_003070) using the analyze particles and time-series analyzer plugins.

### 4.7. Extraction of RNA

Mag Jet RNA Kit (Thermo Fisher Scientific, Waltham, MA, USA) was used for the extraction of total RNA. The RNA’s quality was estimated by electrophoresis in the presence of 1 μg/mL ethidium bromide (2% agarose gel in Tris/Borate/EDTA buffer). The concentration of the extracted RNA was determined with NanoDrop 1000c spectrophotometer. RevertAid H Minus First Strand cDNA Synthesis Kit (Thermo Fisher Scientific, Waltham, MA, USA) was used for reverse transcription of total RNA.

### 4.8. Quantitative Real-Time Polymerase Chain Reaction (RT-qPCR)

Each PCR was performed in a 25 μL mixture composed of 5 μL of qPCRmix-HS SYBR (Evrogen, Moscow, Russia), 1 μL (0.2 μM) of the primer solution, 17 μL water (RNase-free), and 1 μL cDNA. Dtlite Real-Time PCR System (DNA-technology, Moscow, Russia) was used for amplification. The amplification process consisted of the initial 5 min denaturation at 95 °C, 40 cycles of 30 s denaturation at 95 °C, 20 s of annealing at 60–62 °C, and a 20 s extension step at 72 °C. The final extension was performed for 10 min at 72 °C. All sequences were designed with FAST PCR 5.4 and NCBI Primer-BLAST software. The data were analyzed with Dtlite software (DNA-technology, Moscow, Russia). The expression of the studied genes was normalized to gene encoding Glyceraldehyde 3-phosphate dehydrogenase (GAPDH). Data were analyzed using Livak’s method [64].

### 4.9. Statistical Analysis

All presented data were obtained from at least three cell cultures from two to three different passages. All values are given as mean ± standard error (SEM) or as individual cellular signals in experiments. Statistical analyses were performed by paired *t*-test. Differences are significant * *p* < 0.05, ** *p* < 0.01, and *** *p* < 0.001; n/s—data not significant (*p* > 0.05). MS Excel, ImageJ, Origin 2016 (OriginLab, Northampton, MA, USA), and Prism GraphPad 7 (GraphPad Software, RRID: SCR_002798) software were used for data and statistical analysis.

## 5. Conclusions

SeNPs with a diameter of 100 nm have a more pronounced cytoprotective effect on the cells of the cerebral cortex under conditions of ischemia/reoxygenation (OGD/R) through an increased baseline and an OGD/R-induced expression of genes encoding protective proteins, suppression of the OGD-induced global increase in [Ca^2+^]_i_ and activation of reactive astrogliosis. Whereas 50 nm-sized and 400 nm-sized SeNPs, while also exerting a protective effect, regulate the expression of a smaller number of protective proteins and markers of reactive astrogliosis, they do not eliminate the OGD-induced hyperexcitation of neurons, but rather limit the global increase in [Ca^2+^]_i_. One of the explanations for this difference in the effectiveness of the cytoprotective action of different-sized SeNPs is the presence of two endocytosis pathways for large SeNPs (100 and 400 nm) and the presence of some toxic effects in 50 nm-sized SeNPs.

## Data Availability

The data presented in this study are available on request from the corresponding author.

## Supplementary Materials

The following supporting information can be downloaded at: https://www.mdpi.com/article/10.3390/ijms23137464/s1.
