# Supplementary material for: Size-Dependent Cytoprotective Effects of Selenium Nanoparticles during Oxygen-Glucose Deprivation in Brain Cortical Cells
Source: Int J Mol Sci. 2022 Jul 5;23(13):7464. doi: 10.3390/ijms23137464 (PMC9267189; doi:10.3390/ijms23137464)
Supplement: Supplementary file 1 [file ijms-23-07464-s001.zip › ijms-1799821-supplementary.pdf]

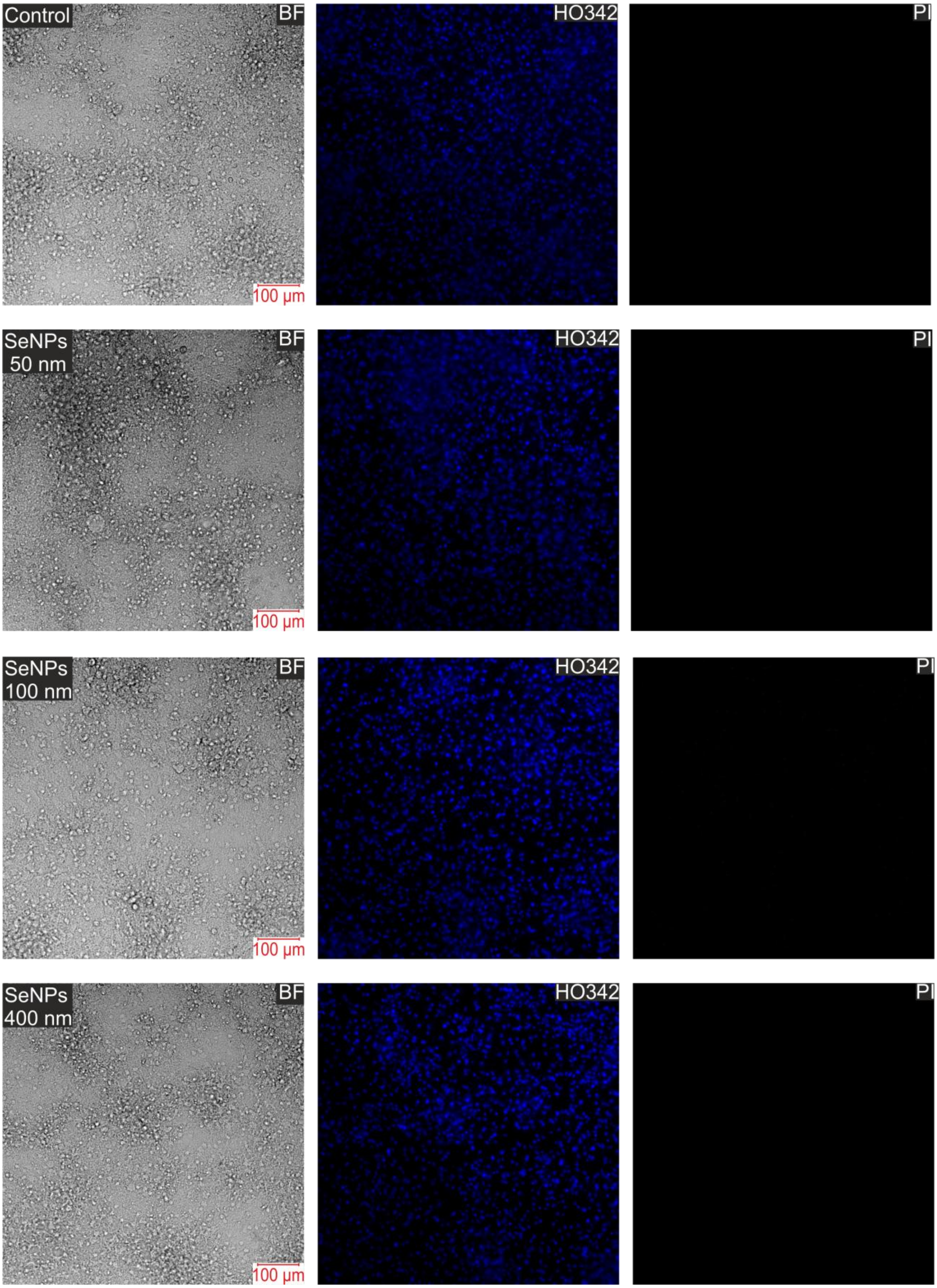

SUPPLEMENTARY, FIGURE S1. The effect of 24-hour incubation of cortical astrocytes with 3 µg/ml of different-sized selenium nanoparticles. Double staining of cells with Hoechst 33342 (HO342), Propidium iodide (PI) and Bright-field microscopy (BF).
